# Supplementary material for: 0.75 Gbit/s high-speed classical key distribution with mode-shift keying chaos synchronization of Fabry–Perot lasers
Source: Light Sci Appl. 2021 Aug 30;10:172. doi: 10.1038/s41377-021-00610-w (PMC8403675; doi:10.1038/s41377-021-00610-w)
Supplement: Supplementary file 1 — Supplementary information [file 41377_2021_610_MOESM1_ESM.docx]

**Supplementary Information for**

**0.75 Gbits^-1^ high-speed classical key distribution with mode-shift keying chaos synchronization of Fabry-Perot lasers**

Hua Gao^1,2^, Anbang Wang^1,2*^, Longsheng Wang^1,2^, Zhiwei Jia^1,2^, Yuanyuan Guo^1,2^, Zhensen Gao^3,4^, Lianshan Yan^5^, Yuwen Qin^3,4^, Yuncai Wang^3,4^

1 Key Laboratory of Advanced Transducers and Intelligent Control System, Ministry of Education and Shanxi Province, Taiyuan 030024, China

2 College of Physics and Optoelectronics, Taiyuan University of Technology, Taiyuan 030024, China

3 School of Information Engineering, Guangdong University of Technology, Guangzhou 510006, China

4 Guangdong Provincial Key Laboratory of Photonics Information Technology, Guangzhou 510006, China

5 Center for Information Photonics and Communications, Southwest Jiaotong University, Chengdu 610031, China

*Corresponding author: [wanganbang@tyut.edu.cn](mailto:wanganbang@tyut.edu.cn)

**S1. Estimation of entropy rate of laser chaos in experiments**

In order to predict the maximum rate of key distribution, we estimated the entropy rate of the experimental laser chaos of mode *λ*_1_ at a wavelength of 1547.768 nm. The entropy rate was estimated as the maximum generation rate of random bits with verified randomness using single-bit quantization method^S1, S2^ with the following process. The chaotic signal was sampled by the real-time oscilloscope and then the sampling points are quantized a binary sequence by comparing their voltages with the median of amplitude distribution. By executing logical XOR between the original binary sequence and its duplicate with a delay of 1.2 ns, a final random bit sequence was achieved. The randomness was examined by the NIST test suite^S3^ using 1000 samples of 1-Mbit sequence with a significance level of 0.01. Figure S1 shows the NIST test results of random bits at different generation rates. Note that, the tests for each generation rate were executed five times, and the median of the numbers of passed items is denoted as black dots, and the maximum and minimum numbers of passed items are labeled with red bars. The random bit sequence is treated as passing the NIST tests when the median equals 15. It is found from Fig. S1 that the random bits with a maximum rate of 16 Gbits^-1^ can pass the tests. This result means that the entropy rate is about 16 Gbits^-1^. The entropy rate of mode *λ*_0_ is the same as mode *λ*_1_ because of the same bandwidth.


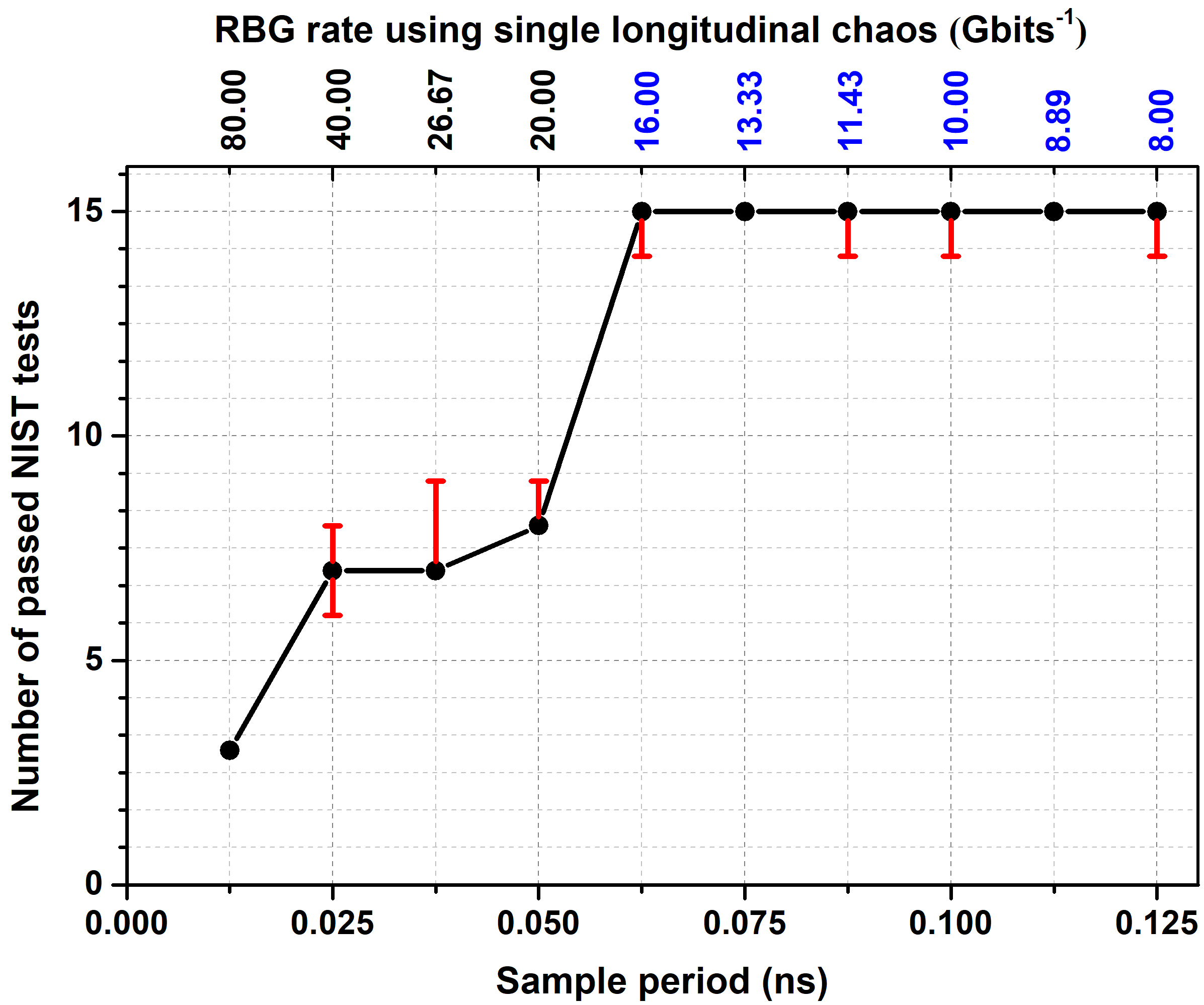


**Fig. S1 Number of the passed NIST test items for random bits at different generation rates.**

**S2. Cross-correlation between outputs of drive source and FP lasers**

We experimentally measured the cross correlation between the drive light and the laser output light both filtered with the same spectral filter with linewidths of 5 nm and 0.83 nm, respectively. As shown in Fig. S2, the cross-correlation values are about 0.34 and 0.38, which are similar to the reported result in ref. S4. The correlation value is greatly lower than the cross-correlation value of about 0.97 between the two lasers with longitudinal mode matching. This means that the information per bit (*I*_E_) known by Eve from the drive light about the common bits of Alice and Bob is far smaller than 1.


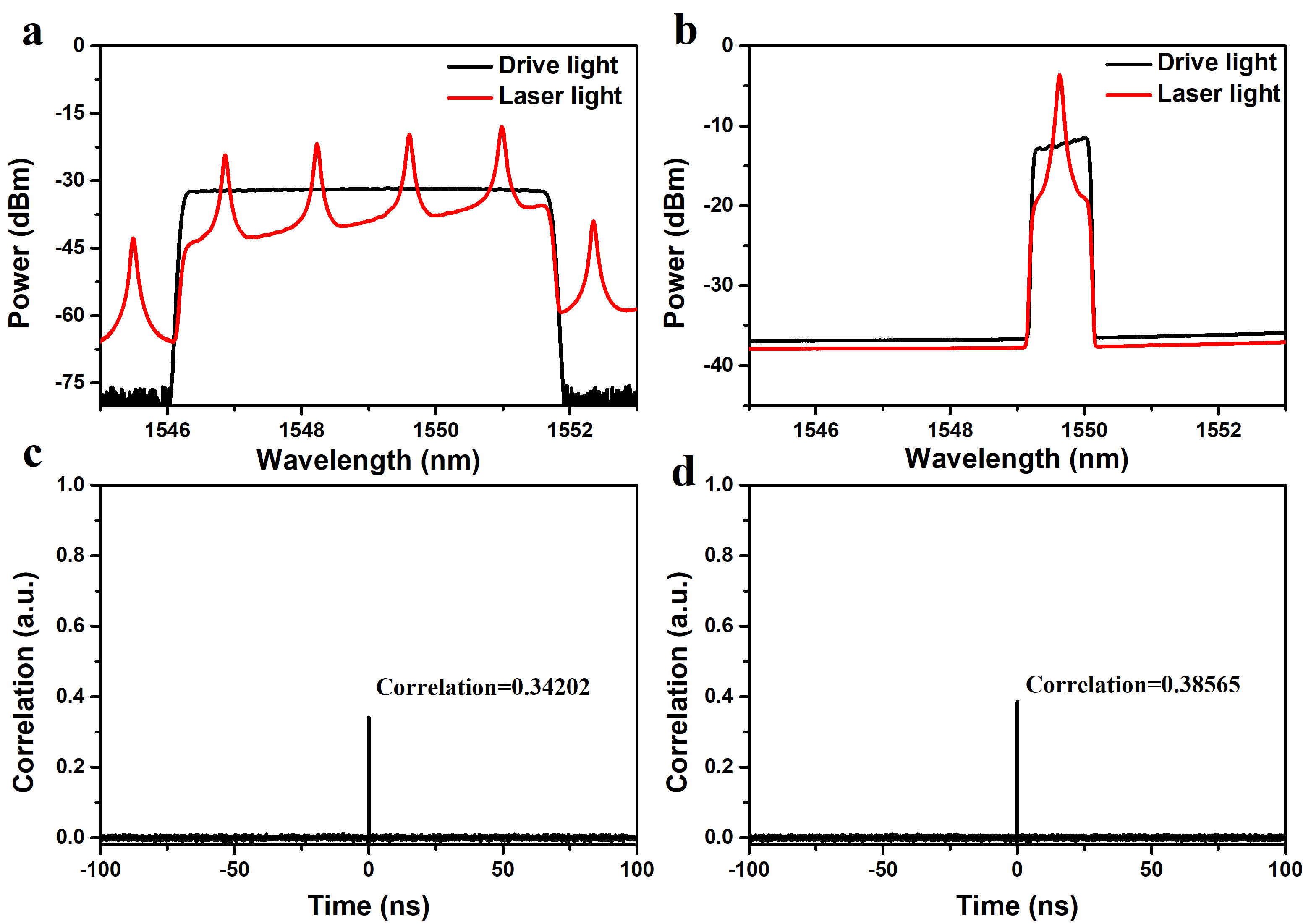


**Fig. S2** **a,b** Measured optical spectra of drive light and laser light filtered with the same spectral filter, and **c,d** the corresponding cross-correlation traces: 5 nm (**a** and **c**) and 0.83 nm (**b** and **d**).

**S3. Long-term stability of chaos synchronization**

In order to verify stability of long-distance synchronization, we recorded the chaotic temporal waveforms within 90 minutes at an interval of 30 seconds, and calculated the cross-correlation. As shown in Fig. S3, an average cross-correlation of 0.9363 with a standard deviation of 0.0009 is obtained, which proves the long-term synchronization stability of the current setup. In addition, we also recorded a 12-minute video of the temporal waveforms of two lasers and the corresponding scatter plot (see supplementary video). The video intuitively demonstrates the synchronization stability of this system with a 160-km fiber link.

It should be mentioned that Koizumi *et al*. reported a loss of synchronization after around 30 seconds in a setup^S5^, in which two optical-feedback semiconductor lasers with a long fiber external cavity are driven by a common light. The loss of synchronization is induced by the variation of optical-feedback phase in the long external cavity. Using a compact photonic integrated external-cavity laser can mitigate the variation of feedback phase and increase stability of synchronization^S7^. By comparison, the lasers used in our setup are monolithic lasers without external feedback cavity, so the perturbation to the optical-feedback phase is avoided. This is the reason why our system can achieve more stable chaos synchronization over the long term.


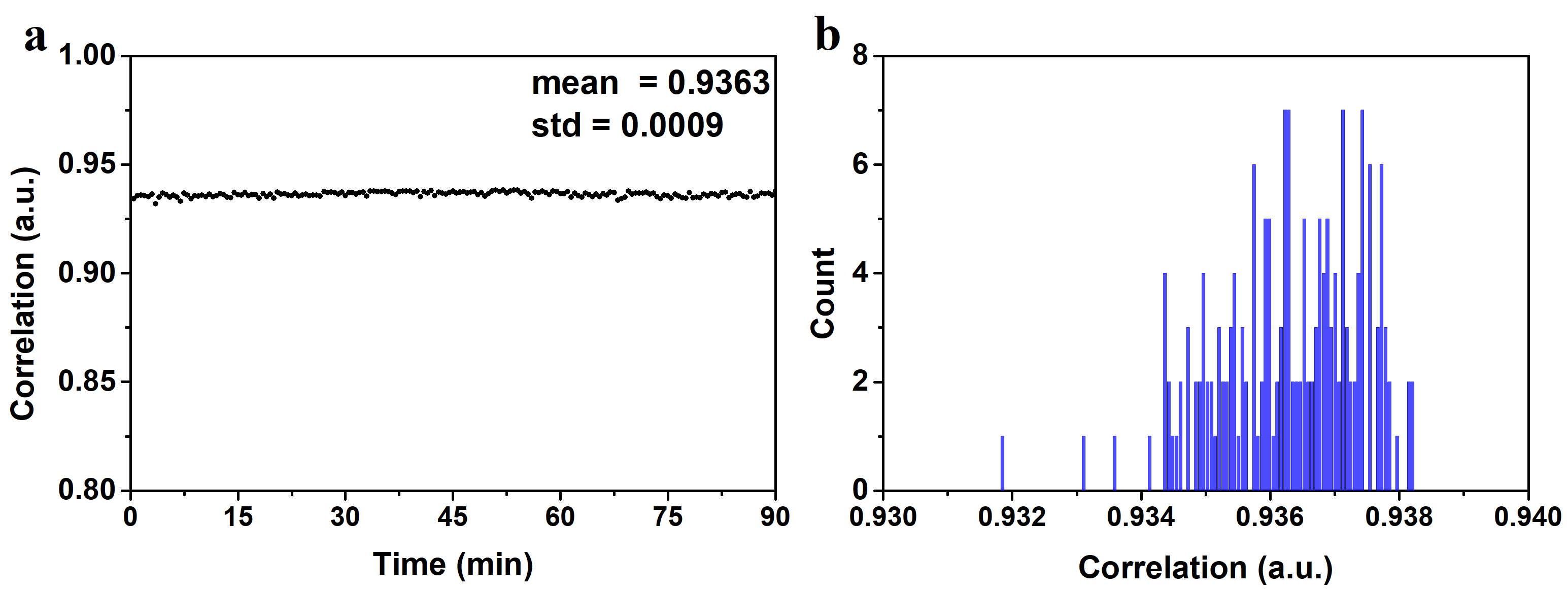


**Fig. S3 a** Measured cross-correlation values vs. time, **b** histogram

**S4. Effects of laser internal parameters on common-signal-induced chaos synchronization**

We numerically simulated the effects of laser internal parameters on chaos synchronization between two Fabry-Perot (FP) lasers driven by an ASE light. The simulation was carried out by VPI Transmission Maker which is a software tool for optical communications. In simulation, a back-to-back configuration of chaos synchronization of two FP lasers similar to the experimental setup was constructed, in order to shorten the calculation time. Essentially, the fiber transmission does not affect the effects of internal parameters on chaos synchronization. First, two FP laser modules with the default settings of parameters were used to achieve chaos synchronization. The internal parameter values of FP lasers are listed in Table S1. The center wavelength and longitudinal mode spacing of the lasers were 1546.79 nm and 0.58 nm, respectively. The injected drive light had a 7-nm spectral width ranging from 1543.3 nm to 1550.3 nm, and the injection power to the lasers was 533 μW. Then, we filtered the mode at 1546.79 nm to investigate the effects of parameter mismatch.

**Table S1. Internal parameter values of FP lasers**

| Parameter | Value | Unit |
| --- | --- | --- |
| Active region length | 650 | *μ*m |
| Active region width | 2.5 | *μ*m |
| Group index | 3.2 | -- |
| Internal loss | 3000 | m^-1^ |
| Interface reflection coefficient | 0.32 | -- |
| Effective mode area | 1.0×10^-12^ | m^2^ |
| Linear gain coefficient | 30×10^-21^ | m^2^ |
| Nonlinear gain coefficient | 1.0×10^-23^ | m^3^ |
| Nonlinear gain time | 0.5 | ps |
| Carrier density at transparency | 1.5×10^24^ | m^-3^ |
| Carrier capture time | 70 | ps |
| Carrier escape time | 140 | ps |
| Initial carrier density | 5×10^23^ | m^-1^ |
| Linewidth enhancement factor | 3 | -- |


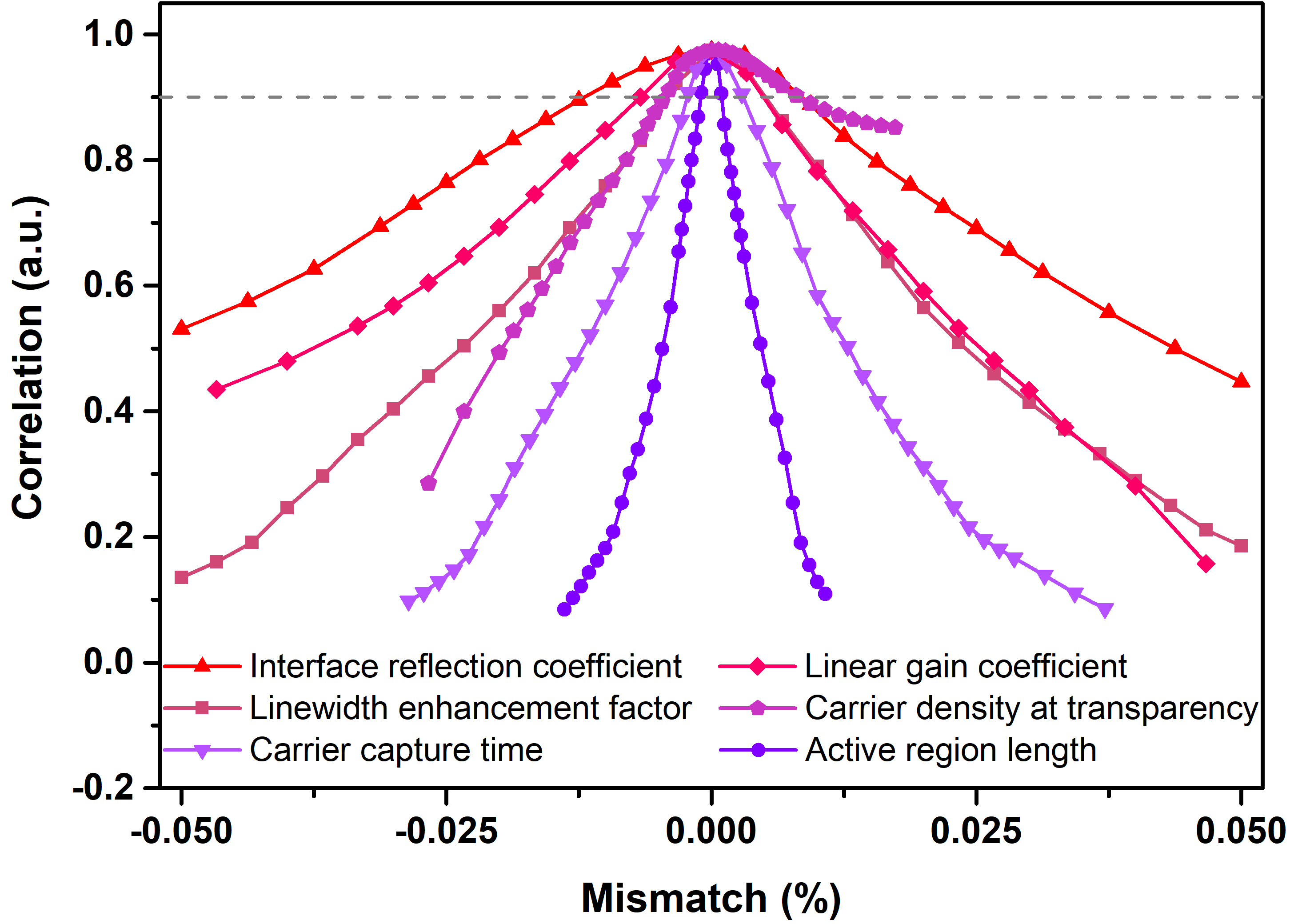


**Fig. S4 Effects of internal parameters mismatch on chaos synchronization**

We mainly took six internal parameters including linewidth enhancement factor *α*, active region length *L*_a_, interface reflection coefficient *R*, linear gain coefficient *g_n_*, carrier density at transparency *N*_0_, and carrier capture time *τ_c_* into account. One of the parameters of laser FP_B_ was adjusted while fixing the others and all the parameters of laser FP_A_. As plotted in Fig. S4, the correlation value reaches a maximum of 0.9474 when all the parameters are matched. We take cross-correlation value of 0.9 as the criteria of the tolerable mismatch range. As a result, the tolerable mismatches of *α*, *L*_a_, *R*, *g_n_*, *N*_0_, and *τ_c_* are −0.41%~0.50%, −0.098% ~ 0.096%, −1.19% ~ 0.85%, −0.60% ~ 0.35%, −0.47% ~ 0.80%, and −0.23% ~ 0.30%, respectively. As reported by Argyris *et al*., only a few matched-pairs out of dozens of lasers prove to be matched with a parameter mismatch below 5% even from the same fabrication wafer^S7^. According to our simulated results, the tolerable mismatches of the common-signal-induced synchronization system are far smaller than 5%. This means that, in order to achieve chaos synchronization, two semiconductor lasers should be selected from the same fabrication wafer.

In addition, according to the tolerable mismatches, we roughly numerically evaluated the lasers’ parameter space using the method reported in ref. S8. The parameter space is estimated at about 10^16^ by considering typical range of parameter values as follows^S9-S14^: *α=*2~7, *L*_a_ = 200~1500 μm, *R* = 0.1~0.9, *g_n_* = 20×10^-21^~160×10^-21^ m^2^, *N*_0_ = 0.2×10^24^~4.2×10^24^ m^-3^, and *τ_c_* = 10 ps ~2 ns. The parameter space will be larger if the other internal parameters are considered. Each combination of hardware parameters in the space means one device. Therefore, it is very hard for Eve to achieve a third laser matched with internal-parameter to achieve synchronization if the users keep the fabrication wafer in private.

**References**

1. Sakuraba, R. *et al*. Tb/s physical random bit generation with bandwidth-enhanced chaos in three-cascaded semiconductor lasers. *Optics Express* **23**, 1470-1490 (2015).
2. Wang, A. B. *et al*. Minimal-post-processing 320-Gbps true random bit generation using physical white chaos. *Optics Express* **25**, 3153-3164 (2017).
3. Bassham, L. *et al*. A Statistical Test Suite for Random and Pseudorandom Number Generators for Cryptographic Applications. (NIST Special Publication, 2010).
4. Tomiyama, M. *et al*. Effect of bandwidth limitation of optical noise injection on common-signal-induced synchronization in multi-mode semiconductor lasers. *Optics Express* **26**, 13521-13535 (2018).
5. Koizumi, H. *et al*. Information-theoretic secure key distribution based on common random-signal induced synchronization in unidirectionally-coupled cascades of semiconductor lasers. *Optics Express* **21**, 17869-17893 (2013).
6. Sasaki, T. *et al*. Common-signal-induced synchronization in photonic integrated circuits and its application to secure key distribution. *Optics Express* **25**, 26029-26044 (2017).
7. Argyris, A. *et al*. Chaos-on-a-chip secures data transmission in optical fiber links. *Optics Express* **18**, 5188-5198 (2010).
8. Wang, D. M. *et al*. Key space enhancement of optical chaos secure communication: chirped FBG feedback semiconductor laser. *Optics Express* **27**, 3065-3073 (2019).
9. Arakawa, Y. & Yariv, A. Quantum well lasers—gain, spectra, dynamics. *IEEE Journal of Quantum Electronics* **22**, 1887-1899 (1986).
10. Nagarajan, R. *et al*. High-speed quantum-well lasers and carrier transport effects. *IEEE Journal of Quantum Electronics* **28**, 1990-2008 (1992)
11. Kikuchi, K. *et al*. Measurement of differential gain and linewidth enhancement factor of 1.5-μm strained quantum-well active layers. *IEEE Journal of Quantum Electronics* **30**, 571-577 (1994)
12. Zhang, Y. J. *et al.* Optimization design of active structure of strained MQW DFB laser, *Chinese Journal of Semiconductors.* **24**, 6-10 (2003)
13. Brox, O. *et al.* High-frequency pulsations in DFB lasers with amplified feedback, *IEEE Journal of Quantum Electronics* **39**, 1381-1387 (2003)
14. Schwertfeger, S. *et al*. High power picosecond pulse generation due to mode-locking with a monolithic 10-mm-long four-section DBR laser at 920 nm, *IEEE Photonic Technology Letters* **19**, 1889-1891 (2007)
